# Multiple heavy metals affect root response, iron plaque formation, and metal bioaccumulation of *Kandelia obovata*

**DOI:** 10.1038/s41598-022-14867-7

**Published:** 2022-08-23

**Authors:** Minwei Chai, Ruili Li, Xiaoxue Shen, Lingyun Yu, Jie Han

**Affiliations:** 1 School of Environment and Energy, Shenzhen Graduate School of Peking University, Shenzhen, 518055 Guangdong China; 2 School of Science and Technology, Hong Kong Metropolitan University, Ho Man Tin, Kowloon Hong Kong, China

**Keywords:** Plant sciences, Ecology, Environmental sciences

## Abstract

Multiple heavy metal pollution in mangrove wetlands is serious. *Kandelia obovata* seedlings were cultured in pots in which lead (Pb), zinc (Zn) and copper (Cu) were added separately and in combinations. The results showed that heavy metal stress improved the rate of root oxygen leakage, enhanced root activity, and reduced root porosity. The root under single heavy metal stress was impacted by the addition of other heavy metals, demonstrating antagonistic or synergistic effects. Iron plaque (IP) formation was improved under single Zn or Cu stress, and inhibited in binary stress of Pb + Cu. The adsorptions of IP on heavy metals in combined stress (Pb, 62–116 μg g^−1^; Zn, 194–207 μg g^−1^; Cu, 35–52 μg g^−1^) were higher than that in single stress (Pb, 18 μg g^−1^; Zn, 163 μg g^−1^; Cu, 22 μg g^−1^). *K. obovata* accumulated higher levels of heavy metals in root (Pb, 7–200 μg g^−1^; Cu, 4–78 μg g^−1^), compared with IP (Pb, 18–116 μg g^−1^; Cu, 22–52 μg g^−1^), stem (Pb, 3–7 μg g^−1^; Cu, 9–17 μg g^−1^), and leaf (Pb, 2–4 μg g^−1^; Cu, 4–7 μg g^−1^). Correlation analysis showed that single and binary stresses affected *K. obovata*, with more significant effect of trinary stress. Regression path analysis showed that multiple heavy metal stress firstly affected root, then indirectly contributed to IP formation, as well as heavy metal in IP and root; at last, heavy metal in IP directly contributed to heavy metal bioaccumulations in root.

## Introduction

Mangrove is important for maintaining ecological balance and biodiversity in coastal zone, being mainly featured with low tide current velocity, high organic matter deposition, and reducibility in sediment^1,2^. To overcome the harsh hypoxia intertidal environment, aerenchyma (root porosity) is developed in mangrove plant to transfer oxygen from aboveground part to root, and release oxygen into the rhizosphere environment, being known as radial oxygen loss (ROL). ROL can affect nutrient uptake and heavy metal tolerance, alter microbial activity and chemical process in the rhizosphere environment, changing the bioavailability of heavy metal through iron plaque (IP) formation on the root surface^3,4^. IP formation acts as “barrier” or “reservoir” to regulate the transfers of heavy metals in plants, being attributed to the status of IP formation^5,6^. On the other hand, IP formation would be affected by heavy metal stress through the following ways: (1) causing oxidative stress and production of peroxide free radical which oxidizes Fe^2+^ to Fe^3+^^7^; (2) altering the ROL and anatomical structure of root (thickened exodermis, enhanced lignification, and reduced aerenchyma)^8^; (3) affecting sediment physic-chemical properties and microbial community to alter the availability of Fe^2+^ in IP formation^9^.

The responses of root and IP formation in terms of single heavy metal stress have been widely reported^10–12^. In actual field environment, several heavy metals coexist normally, and single heavy metal could not really reflect the true occurrence characteristics and biological toxicity of multiple heavy metals due to their complex interactions^13^. Furthermore, combined pollution of heavy metals and other substances could impact the iron plaque formation and heavy metal absorption in plants (Table S1). Owing to urbanization, coastal landfill, and aquaculture, mangroves have inevitably suffered from ubiquitous heavy metals such as Pb, Zn and Cu^14–16^. As for combined heavy metal stress of Pb, Zn, and Cu, the metal tolerance of mangrove species is ascribed to lignification/suberization deposition within root exodermis, which reduces ROL, and delays the uptake of heavy metals in mangrove root^17^. Our previous studies have found that trinary combined Pb, Zn, and Cu could affect physiological characteristics, improve IP formation and heavy metal deposition on the root surface of *Kandelia obovata*, without exploring the interactions among various heavy metals^18,19^. The effects of single, binary, and trinary stress of Pb, Zn and Cu on physiological responses of *K. obovata* are also intensively studied, with synergy and antagonism to be detected^20^. However, the internal influences of apparent responses are not included, especially for heavy metal bioaccumulation. In fact, Zn has the same family, as well as similar ion radius and chemical property with Cd, being more mobile than that of Pb and Cu. Nowadays, the interactive effects of multiple heavy metals in modulating root response, IP formation, and metal adsorption are still ambiguous. It is hypothesized that multiple heavy metals would demonstrate more significant effect than a single metal in affecting root response, thus changing IP formation and heavy metal bioaccumulations. Thus, systematic exploration is conducted on *K. obovata* in regards to single, binary, and trinary metal stresses. This study aims to (1) explore the characteristics of root responses, IP formation and heavy metal bioaccumulation under multiple heavy metals; (2) clarify the interrelations among single, binary, and trinary stresses, as well as the inner links among root responses, IP formation and heavy metal bioaccumulation.

## Results

### Root characteristics of *Kandelia obovata*

**Figure 1:**
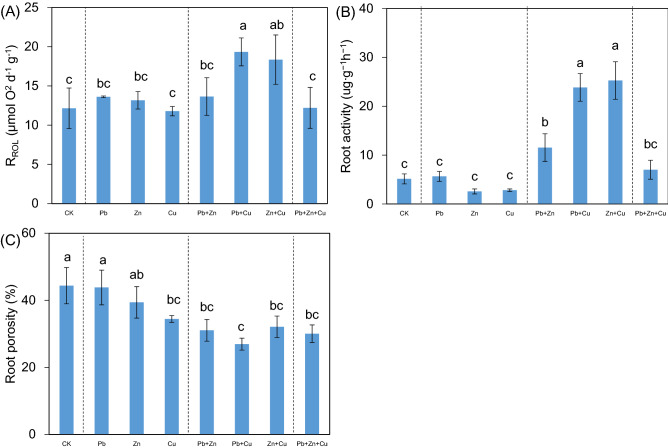
Rate of ROL, root activity, and root porosity of *Kandelia obovata* under individual and different combined stress of Pb, Zn, and Cu. Different lowercase letters indicate significant differences among treatments (*P* < 0.05).

In this study, R_ROL_ did not change significantly in single stress compared to the control (*P* > 0.05, Fig. 1A). Higher level of R_ROL_ was detected in Pb + Cu binary stress compared to single Cu/Pb stress (*P* < 0.05). While, R_ROL_ reduced significantly in trinary stress compared to binary stresses of Pb + Cu and Zn + Cu (*P* < 0.05). In Fig. 1B, no significant change of root activity was detected in single stress (*P* > 0.05), with improved root activity under binary stresses (*P* < 0.05). However, root activity reduced significantly in trinary stress compared to binary stresses of Pb + Cu and Zn + Cu (*P* < 0.05). In Fig. 1C, the root porosity reduced from 44.37 to 34.44% in single Cu stress (*P* < 0.05). Binary stresses of Pb + Zn and Pb + Cu reduced root porosity compared to single Pb stress (*P* < 0.05), with no significant reduce for single Zn or Cu stress (*P* > 0.05). Moreover, no significant change of root porosity was detected in trinary stress compared to binary stresses (*P* > 0.05).

### IP formation and heavy metal immobilizations in *Kandelia obovata*

**Figure 2:**
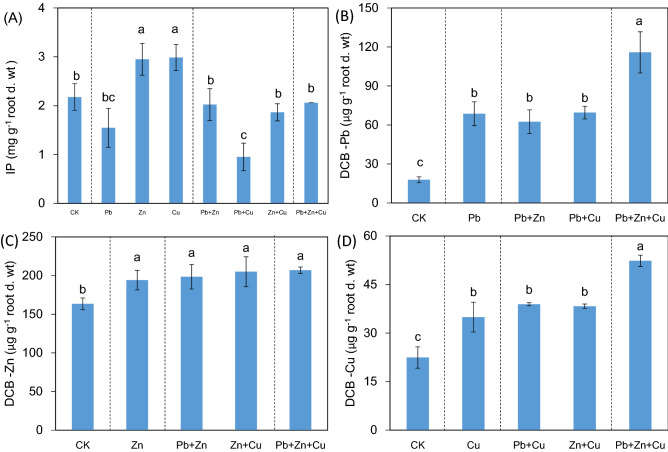
Iron plaque formation on root (mg g^−1^ root d. wt) and concentrations of DCB extractable Pb, Zn and Cu (μg g^−1^ root d. wt) for *Kandelia obovata* under individual and different combined stress of Pb, Zn, and Cu. Different lowercase letters indicate significant differences among treatments (*P* < 0.05).

IP increased significantly under single Zn or Cu stress (*P* < 0.05, Fig. 2A), and binary Zn + Cu stress reduced Fe concentration significantly (*P* < 0.05). Moreover, binary stresses of Pb + Cu and Pb + Zn significantly reduced Fe concentration in single Cu and Zn stress, respectively (*P* < 0.05). Trinary stress significantly improved Fe concentration compared to binary Pb + Cu stress (*P* < 0.05). In Fig. 2B–D, heavy metal stress improved the immobilizations of Pb, Zn, and Cu in IP (*P* < 0.05); No significant change of heavy metal in IP was detected among single and binary stresses (*P* > 0.05); Trinary stress significantly improved the immobilizations of Pb and Cu in IP (*P* < 0.05). In combined stress, heavy metals on IP (Pb, 62–116 μg g^−1^; Zn, 194–207 μg g^−1^; Cu, 35–52 μg g^−1^) were higher than that in single stress (Pb, 18 μg g^−1^; Zn, 163 μg g^−1^; Cu, 22 μg g^−1^).

### Bioaccumulation and transfer of heavy metals in *Kandelia obovata*

**Figure 3:**
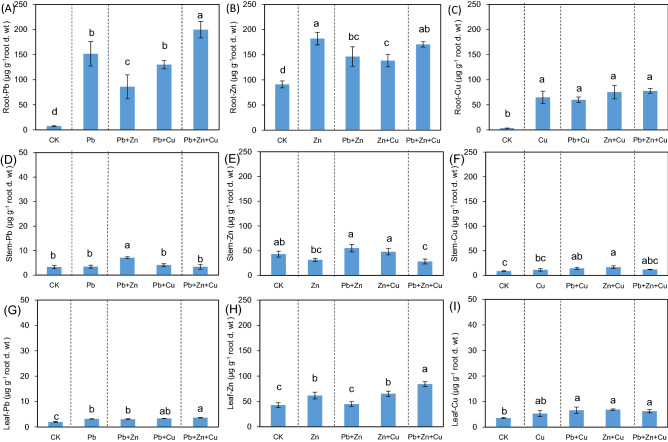
Heavy metals accumulation in roots, stems, and leaves of *Kandelia obovata* under individual and different combined stress of Pb, Zn, and Cu (μg g^−1^ root d. wt). Different lowercase letters indicate significant differences among treatments (*P* < 0.05).

As shown in Fig. 3, the bioaccumulations of heavy metals in roots were higher than that in stem and leaf (*P* < 0.05). *K. obovata* mainly accumulated heavy metals in root (Pb, 7–200 μg g^−1^; Cu, 4–78 μg g^−1^), compared with IP (Pb, 18–116 μg g^−1^; Cu, 22–52 μg g^−1^), stem (Pb, 3–7 μg g^−1^; Cu, 9–17 μg g^−1^), and leaf (Pb, 2–4 μg g^−1^; Cu, 4–7 μg g^−1^). As for Pb in root, Zn reduced Pb concentration in single Pb stress (*P* < 0.05); while, significant improvements were detected in trinary stress compared to binary stresses of Pb + Zn and Pb + Cu (*P* < 0.05). As for Zn in roots, Pb or Cu reduced Zn concentration in single Zn stress (*P* < 0.05), and trinary stress improved Zn concentration in binary Zn + Cu stress (*P* < 0.05). As for Cu in roots, no significant difference was detected among multiple heavy metal stresses (*P* > 0.05). Thus, single heavy metal in *K. obovata* root was affected by the presence of a second or third heavy metal, leading to the inhibited or increased accumulation of heavy metals.

Furthermore, the transfers of heavy metals from IP to root (TF_root_) and from root to leaf (TF_leaf_) indicated that the interactions of heavy metals on TF_root_ levels were limited (*P* > 0.05, Table 1). As for TF_leaf_, though no significant changes of interactive effects were detected for TF_leaf_ levels of Pb and Cu (*P* > 0.05), the presence of Cu in binary and trinary stresses improved TF_leaf_ levels of Zn compared to single Zn stress (*P* < 0.05).

**Table 1:** Transfers of heavy metals from sediments to *Kandelia obovata* under individual and combined stresses.

		CK	Pb	Zn	Cu	Pb + Zn	Pb + Cu	Zn + Cu	Pb + Zn + Cu
Pb	TF_root_	0.42 ± 0.04a	2.21 ± 0.17b			1.40 ± 0.48ab	2.45 ± 1.06b		1.82 ± 0.39b
	TF_leaf_	0.27 ± 0.02b	0.02 ± 0.00a			0.04 ± 0.01a	0.03 ± 0.00a		0.02 ± 0.00a
Zn	TF_root_	0.56 ± 0.03a		0.96 ± 0.02c		0.77 ± 0.14bc		0.68 ± 0.10ab	0.82 ± 0.02bc
	TF_leaf_	0.47 ± 0.02b		0.34 ± 0.04a		0.31 ± 0.05a		0.48 ± 0.08b	0.49 ± 0.03b
Cu	TF_root_	0.16 ± 0.03a			1.85 ± 0.21b		1.54 ± 0.14b	1.97 ± 0.39b	1.49 ± 0.06b
	TF_leaf_	0.99 ± 0.03b			0.08 ± 0.02a		0.11 ± 0.01a	0.09 ± 0.01a	0.08 ± 0.00a

Values are the mean of three replicates and within each line, those not followed the same letters are significantly different (*P* < 0.05).

### Interactions of root response, IP formation, and metal bioaccumulation

In order to explore interactions of plant responses, pearson correlation analysis was performed (Table 2). In this study, IP formation showed negative correlations with root activity (*r* = − 0.643, *P* < 0.01) and R_ROL_(*r* = − 0.531, *P* < 0.05), indicating their indirect even reverse interaction relationships. While, positive correlation was detected among IP formation and root porosity though not significant. Root activity was positively and negatively correlated with root ROL (*r* = 0.758, *P* < 0.01) and porosity (*r* = − 0.536, *P* < 0.01), respectively.

**Table 2:** The correlation coefficients (r) between root growth characteristics and IP formation in *Kandelia obovata.*

	Root porosity	Root activity	R_ROL_	IP formation	Root biomass	Stem biomass	Leaf biomass	Root/Shoot
Root porosity	1							
Root activity	− 0.536**	1						
R_ROL_	− 0.476*	0.758**	1					
IP formation	0.297	− 0.643**	− 0.531**	1				
Root biomass	0.319	− 0.611**	− 0.359	0.645**	1			
Stem biomass	− 0.281	− 0.001	0.015	0.149	0.452*	1		
Leaf biomass	− 0.301	0.068	− 0.073	− 0.051	0.251	0.539**	1	
Root/Shoot	0.560**	− 0.486*	− 0.266	0.437*	0.285	− 0.519**	− 0.772**	1

Shoot = stem + leaf. *, *P* < 0.05. **, *P* < 0.01. *R*_*ROL*_ rate of ROL.

Furthermore, three-way ANOVA analysis indicated that single heavy metal stress affected plant response, except for root ROL, and stem biomass (Table 3). Root activity, root ROL, and IP formation were significantly affected by Pb + Zn (*P* < 0.01). The effects of Pb + Zn, Zn + Cu, and Pb + Cu on heavy metals in IP, root, stem and leaf were significant (*P* < 0.01), except for heavy metal in root under Pb + Cu treatment. There were interactive effects of Pb + Zn + Cu on root porosity, root activity, root ROL, IP formation, root biomass, and heavy metals in IP, root, and stem (*P* < 0.01).

**Table 3:** Three-way analysis of variance of effects Pb, Zn, Cu, and their interaction on biochemical parameters in *Kandelia obovata.*

Source	df	Root porosity	Root activity	R_ROL_	IP formation	Root biomass	Stem biomass	Leaf biomass	Root/Shoot	Heavy metal in IP	Heavy metal in root	Heavy metal in stem	Heavy metal in leaf
Pb	2	4.99*	12.34**	0.95	46.35**	6.42*	0.02	14.95**	18.69**	15.31**	165.24**	15.79**	21.96**
Zn	2	4.13	6.78*	0.02	3.64	0.07	2.43	26.22**	16.87**	452.47**	252.10**	169.15**	905.49**
Cu	2	19.54**	89.90**	7.77*	3.37	1.10	1.90	0.16	2.40	1.01	48.38**	2.47	8.77**
Pb + Zn	4	0.41	73.84**	18.40**	16.57**	4.01	0.07	3.57	0.67	73.41**	9.47**	38.53**	48.83**
Pb + Cu	4	0.27	3.99	0.08	0.08	5.37*	0.96	4.32	0.36	73.37**	52.81**	0.63	152.22**
Zn + Cu	4	4.55*	0.22	0.36	4.55*	0.31	0.01	0.15	0.09	100.89**	47.40**	24.37**	179.51**
Pb + Zn + Cu	8	5.08*	166.63**	12.92**	20.20**	22.47**	2.33	0.13	3.51	18.84**	9.24**	159.47**	4.07

Heavy metals in IP, root, stem, and leaf were the sum of Pb, Zn and Cu in each treatment. *P < 0.05, **P < 0.01, ***P < 0.001.

**Figure 4:**
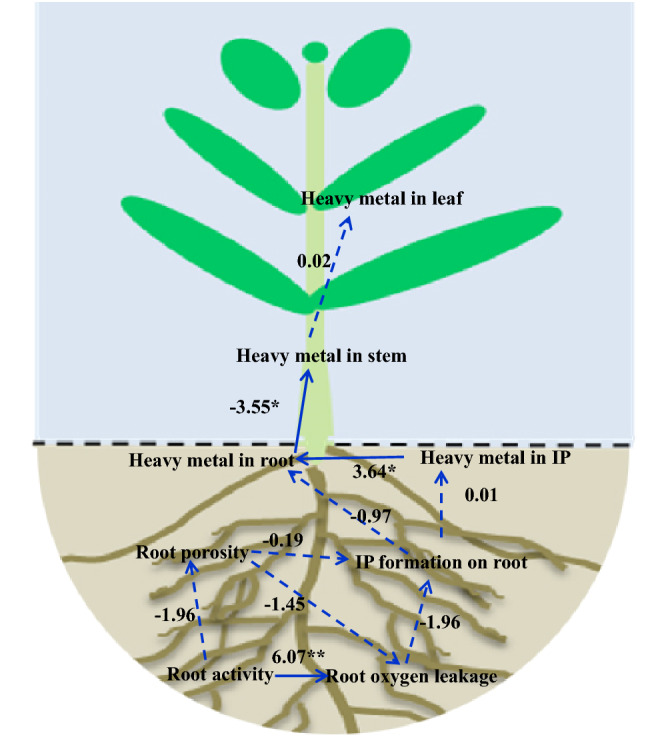
Relationships among root responses, IP formation, and heavy metal bioaccumulation based on RPA. Solid and dashed lines represent the direct and indirect impact, respectively. *P < 0.05. **P < 0.01.

As shown in Fig. 4, the RPA process was conducted among root responses, IP formation and heavy metal bioaccumulation. The results showed that root activity was the main parameter influencing root oxygen leakage (*P* < 0.01). Root porosity and root oxygen leakage did not have direct impact on IP formation, which also did not significantly affect heavy metal in IP and root (*P* > 0.05). Heavy metal in IP significantly affected heavy metals in root (*P* < 0.05), which negatively affected heavy metals in stem (*P* < 0.05).

## Discussion

### Root responses under heavy metal stress

Plant biomass acts as a good indicator for the health of *K. obovata* under heavy metal stress. In the present study, no toxicity symptom in the plant (such as necrosis) was found, indicating higher tolerance of *K. obovata* in responding to multiple heavy metals. Furthermore, the growths of root and stem were not significantly reduced by heavy metals, with leaf to be improved (especially for binary stress of Pb + Zn), leading to higher root/shoot under heavy metal stress (Figure S1). Under multiple heavy metal stress, *K. obovata* was expected to spend extra energy for leaf growth and produce more O_2_, which would be transferred back to the belowground roots to cope with the heavy metal-related effects in rhizosphere environment. In order to explore the interactions of heavy metals on root, various indicators were explored, including root ROL, root activity, root porosity, IP formation, and heavy metal accumulation.

Mangrove plants have evolved aerenchyma in root (being featured with porosity) to adapt to the harsh tidal environment^21,22^. Generally speaking, root porosity in plant (originated from aerenchyma tissue) was regulated by various stimuli, including ethylene and hypoxia^23,24^. Under heavy metal stress, root porosity of mangrove plants would reduce regardless of single or combined heavy metals^25^. In Fig. 1C, root porosity was mainly reduced under heavy metal stress, and no significant interactions were detected among different heavy metal stresses, except for antagonistic effect with binary Pb + Zn stress compared to single Pb stress. Previous study found that root porosity and root ROL were positively correlated in *Bruguiera gymnorrhiza*, and higher level of root porosity facilitated the loss of oxygen from the root to the rhizosphere environment^17^. However, in this study, root porosity of *K. obovata* did not have a similar trend with that of root ROL, and reduced under heavy metal stress, especially for the lowest level in Pb + Cu treatment (Fig. 1A,C). Thus, root porosity was affected not only by single heavy metal application, but also by their combinations.

As for mangrove plants *Avicennia marina*, *Aegiceras corniculatum*, and *B. gymnorrhiza*, the combined stress of Pb, Zn and Cu inhibited seedling growths and reduced root ROL^26^. Furthermore, under combined stress of Pb, Zn and Cu, the reduced root ROL in *B. gymnorrhiza* coexisted with metal-induced inhibition of growth and root aeration, which may be, at least partly, related to the damage to root tissues such as aerenchyma^17^. However, the improved levels of root ROL were detected under binary stresses of Pb + Cu and Zn + Cu (Fig. 1), demonstrating plant species-specific characteristics in response to heavy metal stresses. As for root ROL and root activity, there were synergistic effects with binary stress of Pb + Cu compared to corresponding single metal stress, with the same effect for root activity under binary stress of Zn + Cu; while, antagonistic effects were detected in trinary stress of Pb + Cu + Zn compared to binary stresses of Pb + Cu and Zn + Cu (Fig. 1). The improved root ROL under heavy metal stress was expected to be beneficial for maintaining the oxidative condition, reducing the combination among metal and sulfur, and increasing the bioavailability of heavy metals. Under heavy metal stress, *K. obovata* would mainly promote root activity to increase root ROL, not by increasing root porosity (Fig. 1). The possible explanation may be that at least part of oxygen was transported to belowground roots for aerobic metabolism instead of spreading into the rhizosphere environment, which deserved further investigation.

### IP formation under heavy metal stress

Generally speaking, IP formation is an adaptive behavior of plants to stressful environment^11,27^. Previous studies have found that ROL in plant root was important in affecting IP formation, and heavy metal stress inhibited oxygen release from roots to reduce IP formation^10,28^. Heavy metals affected IP formation in wetland plants, with single Cd to improve IP formation in *K. obovata* and *Avicennia marina*^4,7^. In this study, Pb reduced IP formation under single Zn or Cu stress, and Zn improved IP formation in binary stress of Pb + Cu (Fig. 2A). Thus, the interactions of heavy metals on IP formation were heavy metal type and combination-specific to some extent. On the other hand, the response of IP formation was not similar to that of root ROL, root activity, and root porosity (Fig. 1), which was also verified by their insignificant (*r* = 0.297, *P* > 0.05) or negative (*r* = − 0.643, − 0.531, *P* < 0.01) correlations (Table 2). In particular, the negative correlation between IP formation and root ROL in this study may be due to the formation of oxygen permeation barrier affecting root ROL^29^. Yang et al. (2012) also found that the highest level of root ROL and lowest thickness of IP coexisted in *Veronica serpyllifolia*^30^. Thus, it was expected that the roles of root ROL, root activity, and root porosity on IP formation of *K. obovata* were limited, and some other factors should be investigated further. In fact, apart from root ROL, there were also some biological and abiotic factors affecting IP formation, including microorganism, root exudate, Fe^2+^ activity, and soil moisture^31,32^.

### Heavy metal bioaccumulation under heavy metal stress

Nowadays, the role of IP on heavy metal absorption and transfer in plants is inconclusive, with IP to promote or prevent the absorption of heavy metals in rhizosphere environment^5,12,33^. This study showed that IP formation was only improved under single Zn or Cu stress, the heavy metals immobilized in IP were all improved, and trinary stress improved the immobilizations of Pb and Cu (not Zn) compared to binary stress (Fig. 2). Huang et al. (2012) also reported that IP formation on plant root especially for rice would age/decompose during the whole growth process^34^. As for wetland plants, low IP formation coexisted with high root activity^35^. Root activity was negatively correlated with IP formation (Table 2). Thus, it was not always that more IP formation coexisted with more heavy metal immobilization, which would be affected by IP vitality, especially for higher root activity under binary stresses of Pb + Cu and Zn + Cu.

IP can combine with heavy metals and nutrients by adsorption and co-precipitation, affecting their distribution and accumulation in plants^5,19,36^. As showed in Fig. 3 and Table 1, heavy metals were mainly distributed in roots (especially for Pb and Cu), which were also verified by their lower levels of TF_leaf_. The phytostabilization of Zn in root was limited, with more distribution to be detected in stem and leaf compared to Pb and Cu (Fig. 3), which was also verified by higher TF_leaf_ of Zn (Table 1). In fact, the similar physic-chemical properties of Zn with highly mobile Cd (such as valence state and iron radius) resulted to the absorption and transfer of Zn into the aboveground parts^13,37,38^. Under combined heavy metal stress, IP formation affected the transfer of Pb from sediment to rice, instead of Cd or Cu^39^. As for Pb and Zn in roots (Fig. 2), binary stress demonstrated antagonistic effect compared with single stress, and trinary stress resumed heavy metal accumulation compared to binary stress. While, such trends were not significant for Cu accumulated in root. As for allocation strategies of heavy metals between IP and root (Table 1), Cu and Pb were mainly distributed in roots with high TF_root_ levels (> 1), and Zn mainly accumulated in IP with lower TF_root_ levels (< 1). Thus, the interactions of heavy metals on allocation strategies between IP and root were limited.

In order to fully explore the interactive effects of single, binary, and trinary heavy metals, pearson correlation analysis was performed (Table 2). The results indicated that single and binary stresses had impact on plant responses to some extent, with more significant effect of trinary stress. Furthermore, RPA process was conducted to comprehensively explore root responses, and variations of IP formation and heavy metal bioaccumulations caused by multiple heavy metals (Fig. 4). PRA process was widely applied in exploring relationships among various parameters from an overall perspective^40–42^. In this study, multiple heavy metal stress firstly affected root responses, which indirectly contributed to IP formation on root, as well as heavy metal accumulations in IP and root; while heavy metals in IP directly contributed to their bioaccumulations in root, reducing their transfers to aboveground parts.

## Conclusions

This study mainly explored the interactions of Pb, Zn, and Cu on root growth, IP formation, and heavy metal bioaccumulation in *Kandelia obovata*. The results showed that the root ROL, root activity, root porosity, and IP formation under heavy metal stress were affected with the presence of other metals. Furthermore, the changes of root ROL and root activity were not inhibited under the presence of heavy metals, being different from the reducing root porosity. Thus, heavy metal stress would change root ROL through affecting root activity of *K. obovata*. The adsorption of IP on heavy metals in combined stress was higher than that in single stress. Most heavy metals were stabilized in belowground parts (especially for Pb and Cu), with limited interactions of heavy metals on their allocations between root and IP. Furthermore, the phytoextraction of Zn in leaf was higher than that of Pb and Cu, which was improved with the presence of Cu in binary and trinary stresses. Single and binary heavy metal stresses affected plant responses, with more significant effect to be detected in trinary stress. Overall, multiple heavy metals affected root responses, indirectly impacted IP formation, and heavy metals in IP and root, and directly impacted on heavy metal in plant, especially for root.

## Methods

### Materials preparation and treatments

#### Materials preparation

The propagules of *K. obovata* and sediment for plant culture were collected from mangrove wetlands in Futian National Nature Reserve, Shenzhen, China (114° 00′–114° 02′ E, 22° 30′–22° 32′ N). In details, propagules (20 cm tall with no fungi infections and insect damages) were planted into the seedbed (50 × 40 × 15 cm, length, width, height) filled with clean sand. The seedbed was irrigated with 1/2-strength Hoagland’s nutrient solution (500 mL, 5‰ NaCl, pH 6.5). During the experiment, the deionized water was provided to ensure the moist every morning as previous reports^19,20^. After 2 months, the seedlings were prepared for pot experiment with combined heavy metal stresses.

#### Experiment design

The sediment matrix was mixed fully and separated into 8 groups (Table 4), including control, Pb, Zn, Cu, Pb + Zn, Pb + Cu, Zn + Cu, and Pb + Zn + Cu. The background physicochemical properties of sediment matrix were: moisture, 61.2%; Eh, –204.2 mV; pH, 6.7; salinity, 13‰; TOC, 5.5%; EC, 21.3 mS cm^–1^; Pb, 88.55 μg g^−1^; Zn, 216.03 μg g^−1^; Cu, 65.53 μg g^−1^^19,20^. PbCl_2_, ZnCl_2_, and CuCl_2_ were applied into sediments based on the actual heavy metal pollution status in mangrove wetlands^17,43^. Chemical reagents (guaranteed reagent, GR) were firstly dissolved in deionized water, and then homogenized with the sediment matrix. The concentrations of Pb, Zn and Cu in different treated sediment matrix were presented in Table 4. Furthermore, the sediment matrix was kept fresh by irrigating deionized water and mixed every week^19,20^. After 2 months, the sediment matrix was used for pot experiments. A total of 32 pots were used (19.0 × 18.0 cm, diameter, height). In each pot, air-dried sediment (3 kg) was filled in and a nylon net (16.0 × 16.0 cm length, width; 500 mesh) was installed. Three uniform seedlings of *K. obovata* were transplanted into the nylon net installed in the pot. The nylon net could restrict root growth and help the roots to be sampled from sediment^44^.

**Table 4:** Mixture matrix design of the mixture of Pb, Zn, and Cu in the this study.

Number	Treatment groups	Designed concentrations (μg g^−1^)			Actual concentrations (μg g^−1^)			
		Pb	Zn	Cu	Pb		Zn	Cu
1	Control	0	0	0	62		193	45
2	Pb	400	0	0	363		206	47
3	Zn	0	600	0	56		602	46
4	Cu	0	0	400	65		207	416
5	Pb + Zn	400	600	0	388		696	48
6	Pb + Cu	400	0	400	375		207	414
7	Zn + Cu	0	600	400	92		829	448
8	Pb + Zn + Cu	400	600	400	491		800	454

#### Management

All the experimental pots were placed outdoors randomly and were protected from the rain by a transparent canopy. The temperatures in the summer and autumn were 26–32 °C and 22–31 °C, respectively. The pots were irrigated with deionized water to compensate for evaporation loss of water every morning^19,20^. The plant culture and experimental implementation were shown in Figure S2.

### Sampling and determination

After 5 months, the seedlings of *K. obovata* were collected, which were firstly separated (roots, stems, and leaves) and then dried at 70 °C for two days to obtain the constant weight. Some fresh roots were used to determine root ROL, root activity, root porosity, and IP formation. The root activity was determined by triphenyl tetrazolium chloride (TTC), which was commonly used in studies on plant stress^45^.

### Determination of root ROL

ROL was determined colorimetrically^22,46^, which can be expressed as follows:

$$ {\text{Rate of ROL(R}}_{{{\text{ROL}}}} {\text{) = c(y}} - {\text{z)/g}} $$

where rate of ROL (R_ROL_) is the rate of radial oxygen loss (μmol O_2_ kg^−1^ root d.w. h^−1^); *c* is the initial volume of Ti^3+^-citrate added to each tube (L); *y* is the concentration of Ti^3+^-citrate solution of control (without plants) (μmol Ti^3+^ L^−1^); *z* is the concentration of Ti^3+^-citrate solution (μmol Ti^3+^ L^−1^); *g* is root dry weight after drying at 70 °C for 72 h (in kg).

### Determination of root porosity

Root porosity (% gas volume/root volume) was measured for the entire lateral roots by a pycnometer method^47,48^, which can be expressed as follows:

$$ {{Root\;porosity}}(\% ) = \frac{{\left( {FA - FB} \right)}}{FW + TW - FB} \times 100, $$

where *FA* is the mass of pycnometer with water and vacuumed roots; *FB* is the mass of pycnometer with water and fresh roots in g; *FW* is the mass of water-filled pycnometer in g; *TW* is the mass of fresh roots in g.

### Determination of iron plaque and heavy metals

The cold dithionite–citrate–bicarbonate (DCB) solution was used to extract IP from the root surface within 24 h after harvesting^22,49^. The dry roots, stems, and leaves were grounded and digested with HNO_3_/HClO_4_ (10:1, v/v, USEPA, 1996)^50^. IP was determined using an atomic absorption spectrophotometer (TAS-990, China), with Pb, Zn, and Cu immobilized in IP, plants, and sediments to be determined using inductively coupled plasma-atomic emission spectrometry (Optima 2000 DV, Perkin Elmer, USA). IP was expressed as 0.1591 × [Fe^n+^] in the extraction/root dry weight (mg g^−1^ root d‧wt). The detection limits for Fe, Pb, Zn, and Cu were 0.002, 0.001, 0.003, and 0.003 μg mL^−1^, respectively. The reagents with no sample addition were regarded as blank. Furthermore, internal standard method was used to test the recovery of heavy metals in samples. In this study, the recoveries of heavy metals ranged from 94.45% to 107.21%. The translocation factors of heavy metals in plant were expressed as: TF_root_ = C_root_/C_DCB_, TF_leaf_ = C_leaf_/C_root_. C_root_, C_leaf_, and C_DCB_ are heavy metal concentrations in root, leaf, and DCB extract, respectively.

### Statistical analysis

The data were shown as means ± standard deviation (S.D.) with triplicates. One-way ANOVA and Duncan test were conducted to determine the significant difference among different groups. A three-way ANOVA was conducted to assess interactive effects of different heavy metal stress on root growth, iron plaque formation, and metal bioaccumulation. Pearson correlation analysis was performed to explore the relationships among different growth parameters. Regression path analysis (RPA) was employed to identify relationships among root response, IP formation, and heavy metal bioaccumulations. In this study, SPSS Version 20.0 (IBM Inc, USA) was used to perform all statistical analysis.

### Research statement

Experimental research on plants complies with relevant institutional, national, and international guidelines and legislation. Appropriate permission was obtained as plant samples were collected in this study.

## Supplementary Information

Supplementary Information.
