# Supplementary material for: Multiple heavy metals affect root response, iron plaque formation, and metal bioaccumulation of Kandelia obovata
Source: Sci Rep. 2022 Aug 23;12:14389. doi: 10.1038/s41598-022-14867-7 (PMC9399144; doi:10.1038/s41598-022-14867-7)
Supplement: Supplementary file 1 — Supplementary Information. [file 41598_2022_14867_MOESM1_ESM.docx]

**Figure S1** The growth of *Kandelia obovata* (root, stem, leaf, and root/shoot) under individual and different combined stress of Pb, Zn, and Cu. Different lowercase letters indicate significant differences among treatments (*P* < 0.05). Shoot=tem+leaf.


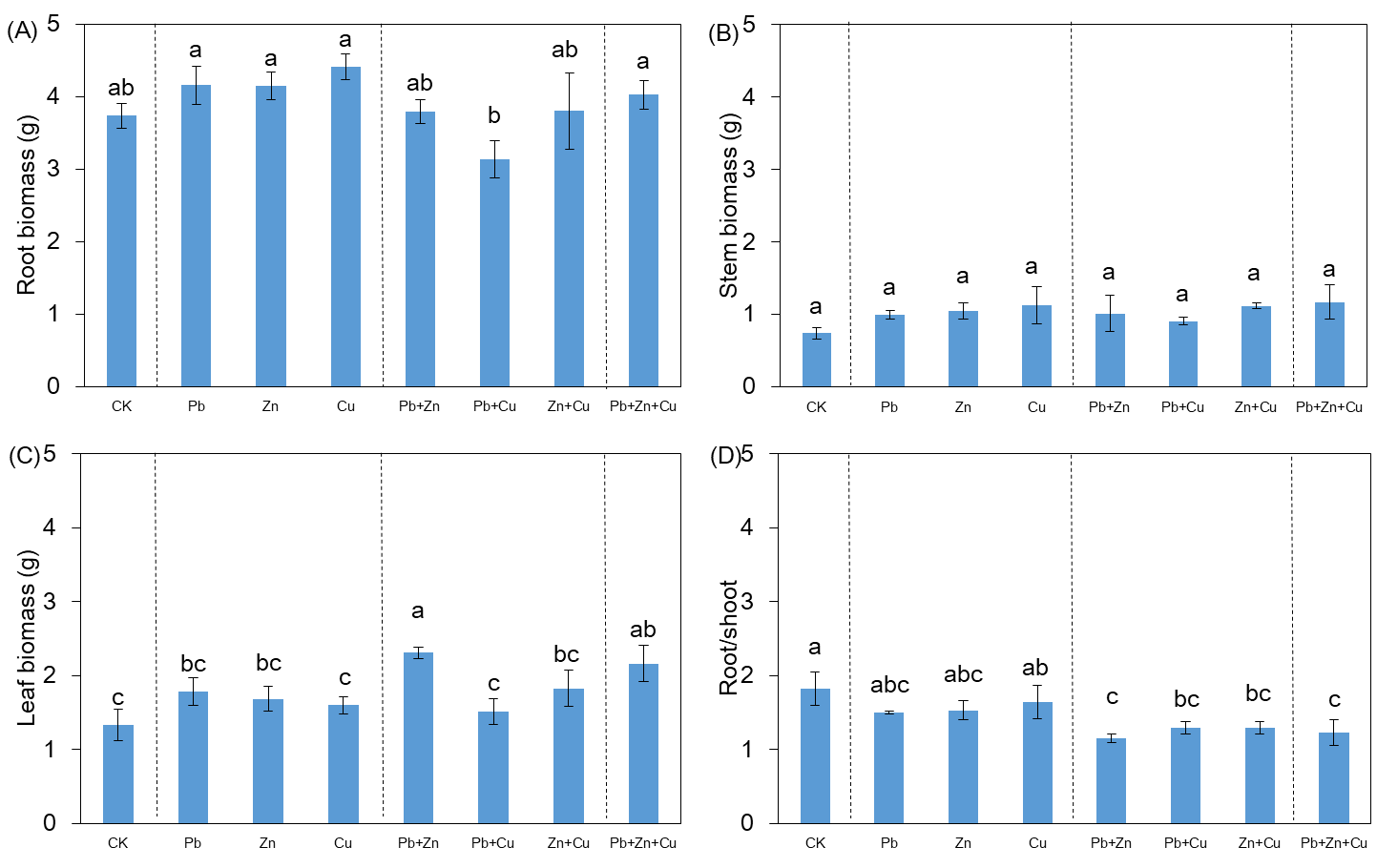


**Figure S2** Plant cultivation and experiment implication


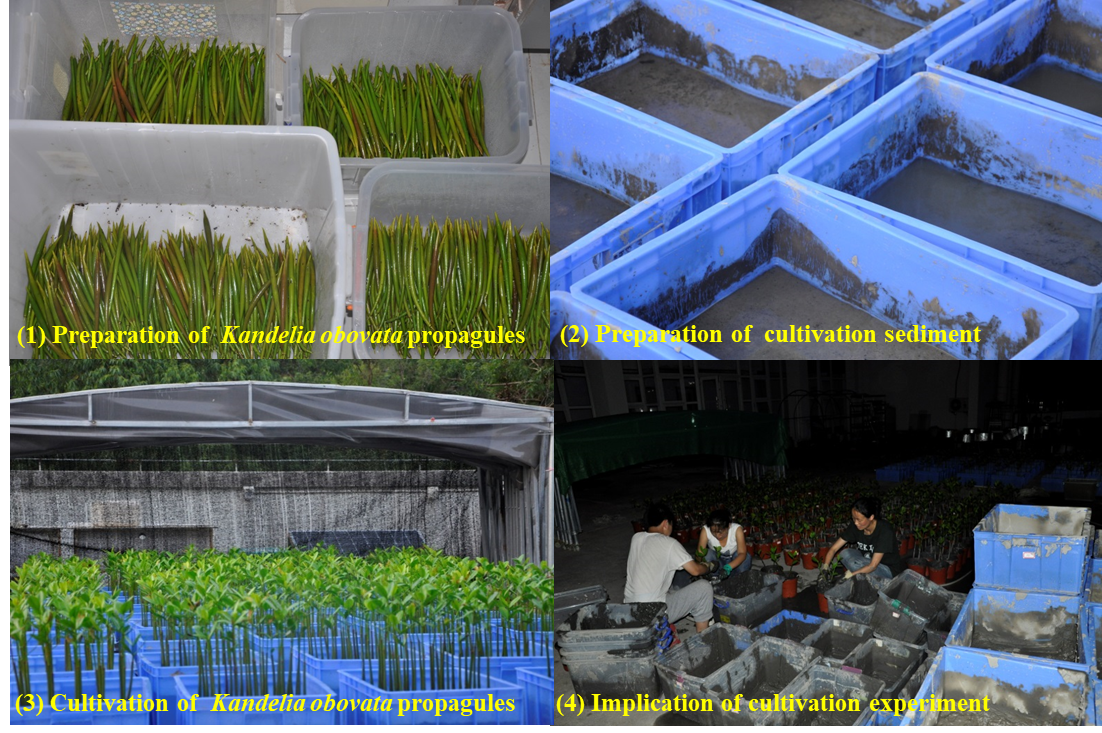


**Table S1** Effects of combined pollution on root surface iron plaque and its function in absorption of heavy metals in plants

| Elements | Plant | Interaction | References |
| --- | --- | --- | --- |
| Zn, P+Cd | *Oryza sativa* | Zn reduced Cd bioaccumulation in root, and improved Cd bioaccumulation in aboveground part; P had no impact on Cd in iron plaque, and improved Cd bioaccumulation in plant. | Liu et al., 2007 |
| S, Cd | *Oryza sativa* | The impact of S on iron plaque formation was limited.  S reduced Cd adsorption under higher Cd stress, and improved Cd adsorption under lower Cd stress. | Gao et al., 2010 |
| S, Cd | *Oryza sativa* | S reduced iron plaque formation and Cd adsorption in grain, but improved Cd adsorption in root. | Fan et al., 2010 |
| Se, Hg | *Oryza sativa* | The single or combined action of iron plaque and Se significantly reduced the adsorption and transfer of Hg in *O. sativa*. | Li et al., 2014 |
| Se (VI), Cd (Fe) | *Oryza sativa* | Se andiron plaque reduced Cd adsorption in plant. | Chen et al., 2014 |
| Pb, Fe | *Carex cinerascens* | The interaction of Fe and Pb significantly impacted Pb in iron plaque. Pb in iron plaque increased with increasing Pb concentration in soil. | Liu et al., 2015 |
| S, Pb | *Oryza sativa* | S reduced the bioavailability of Pb, and impacted iron plaque formation. | Yang et al., 2016 |
|  |  |  |  |
| Cd, Pb | *Phytolacca americana* | Pb impacted Cd accumulation in iron plaque, which was different in terms of iron plaque formation | Cai et al., 2017 |
| P, Cd | *Kandelia obovata* | Combined Cd and Pb improved iron plaque formation.  P improved Cd deposition in iron plaque, and Cd bioaccumulation in roots, and reduced the transfer of Cd to aboveground part. | Dai et al., 2017 |

Cai, C.T., Tang, K.L., Xu, X.P., & Li, F.Y. Effects of iron-manganese plaque thickness on Cd accumulation in *Phytolacca Americana* under Cd-Pb combined stress. Acta Scientiae Circumstantiae, 37, 298-307 (2017).

Chen, M.X., Cao, L., Song, X.Z., Wang, X.Y., Qian, Q.P., & Liu, W. Effect of iron plaque and selenium on cadmium uptake and translocation in rice seedlings (*Oryza sativa*) grown in solution culture. Int. J. Agric. Biol., 16, 1159-1164 (2014).

Dai, M.Y., Liu, J.C., Liu, W.W., Lu, H.L., Jia, H., Hong, H.L., & Yan, C.L. Phosphorus effects on radial oxygen loss, root porosity and iron plaque in two mangrove seedlings under cadmium stress. Mar. Pollut. Bull., 119, 262-269 (2017).

Fan, J.L., Hu, Z.Y., Ziadi, N., Xia, X., & Wu, C.Y.H. Excessive sulfur supply reduces cadmium accumulation in brown rice (*Oryza sativa* L.). Environ. Pollut., 158, 409-415 (2010).

Gao, M.X., Hu, Z.Y., Wang, G.D., & Xia, X. Effect of elemental sulfur supply on cadmium uptake into rice seedlings when cultivated in low and excess cadmium soils. Commun. Soil Sci. Plan., 41, 990-1003 (2010).

Li, Y.Y., Zhao, J.L., Gao, Y.X., Li, Y.F., Li, B., & Zhao, Y.L., et al.. Effects of iron plaque and selenium on the absorption and translocation of inorganic mercury and methylmercury in rice (*Oruza sativa* L.). Asian J. Ecotoxicol., 9, 972-977 (2014).

Liu, C.Y., Chen, C.L., Gong, X.F., Zhou, W.B., & Yang, J.Y. Progress in research of iron plaque on root surface of wetland plants. Acta Ecologica Sinica, 34, 2470-2480 (2014).

Liu, H.J., Zhang, J.L., Christie. P., & Zhang, F.S. Influence of external zinc and phosphorus supply on Cd uptake by rice (*Oryza sativa* L.) seedlings with root surface iron plaque. Plant Soil, 300, 105-115 (2007).

Yang, J.X., Liu, Z.Y., Wan, X.M., Zheng, G.D., Yang, J., & Zhang, H.Z., et al. Interaction between sulfur and lead in toxicity, iron plaque formation and lead accumulation in rice plant. Ecotox. Environ. Safe., 128, 206-212 (2016).
